# The Moderating Effect of Sex on Autistic Trait Emotional Intelligence, Alexithymia, and Empathy

**DOI:** 10.1007/s10803-024-06540-x

**Published:** 2024-09-26

**Authors:** Mary Isaac Cargill, Matthew D. Lerner, Erin Kang

**Affiliations:** 1 Psychology Department, Montclair State University, 1 Normal Ave, Montclair, NJ 07043 USA; 2 AJ Drexel Autism Institute, Drexel University, 3020 Market Street Suite #560, Philadelphia, PA 19104 USA

**Keywords:** Autism, Adults, Sex differences, Emotional intelligence, Alexithymia, Empathy

## Abstract

Autism spectrum disorder (ASD) is associated with differences in social communication, and these differences are related to trait emotional intelligence (TEI), alexithymia, and empathy. Autism is known to present differently in males and females, but research on sex differences in TEI, alexithymia, and empathy is largely relegated to non-autistic people. Therefore, the current research sought to explore individual relationships between autistic characteristics and TEI, alexithymia, and empathy, as well as the possible influence of sex in these relationships. In the current study, autistic and non-autistic adults reported on their autistic characteristics, TEI, alexithymia, and empathy. Based on previous research, it was hypothesized that more autistic characteristics would be associated with less TEI, more alexithymia, and less empathy, and that these relationships would be more prominent amongst males. More autistic characteristics were associated with greater challenges across the three areas of interest. However, only the relationship between TEI and autistic characteristics was moderated by sex, such that males demonstrated higher support needs related to TEI than females. Results from this analysis indicate that adults with more autistic characteristics, regardless of diagnostic status, demonstrate differences in TEI, alexithymia, and empathy. The current analysis may offer additional context to the evolving understanding of empathy and autism by suggesting that TEI and alexithymia could account for differences in empathy. Moreover, sex seems to play a role in the relationship between autistic characteristics and TEI such that differences are especially prominent for males.

Autism spectrum disorder (ASD) is a lifelong neurodevelopmental disorder characterized by differences in social communication and the presence of specific patterns of behaviors and interests (American Psychiatric Association, 2013). Autism’s presentation is incredibly heterogenous – there are a myriad of facets (e.g., sensory sensitivities, co-occurring mood disorders, sleep problems, gastrointestinal issues, etc.) associated with autism in addition to the core diagnostic characteristics (Kerns & Kendall, 2012; Madra et al., 2020; Richdale & Schreck, 2009; Hazen et al., 2014). As a result of this heterogeneity, support needs vary widely both between and within autistic^1^ people. Additionally, a growing body of evidence suggests that autism presents differently in males and females such that males may have greater social play abilities and more specific patterns of interests and behaviors than females (Rivet & Matson, 2011; Ferri et al., 2018). Social communication is also thought to differ between the sexes, however the nature of this difference is presently unclear (Rivet & Matson, 2011). Furthermore, the differential social communication that is characteristic of autism is uniquely related to trait emotional intelligence (TEI), alexithymia, and empathy (Boily et al., 2017; Gerber et al., 2019; Hill, 2009). TEI, alexithymia, and empathy have each been the focus of autism research, with findings indicating that co-occurring alexithymia is related to differences in empathy and components of TEI in autistic adults (Bird et al., 2010; Cook et al., 2013). Given the relationships suggested between alexithymia, TEI, and empathy, joint examination of these constructs in autistic adults is warranted, as these seem to be underlying facets of the social difficulties that autistic adults experience. While previous work has examined these constructs individually, there is currently a gap in the literature regarding concurrent consideration of these related areas and their associations to the level of autistic traits. Furthermore, research in these areas has largely neglected to consider sex differences.

## Emotional Intelligence

Emotional intelligence refers to a person’s abilities and the facets of their personality that enable them to cope with their own emotions and others’ emotions. Emotional intelligence can be conceptualized in two models: the ability emotional intelligence model, and the trait emotional intelligence model. Ability emotional intelligence (AEI) refers to people’s cognitive ability to perceive, interpret, understand, and utilize emotional information (Mayer et al., 2004). AEI is typically measured in a manner similar to intelligence wherein scores are determined based on correct responses to a set of questions or scenarios (Petrides, 2011). Alternatively, trait emotional intelligence (TEI) describes a personality trait reflected in self-perceptions of their own emotional intelligence as the factors that affect how one interacts with emotional information (Pardeller et al., 2017). Whereas AEI is typically measured by tests that assess an individual’s maximum ability by judging their performance as either correct or incorrect, TEI is measured via self-report questionnaires intended to gauge subjective self-perceptions of emotional abilities (Petrides, 2011). While there is evidence to suggest that autistic people have largely intact AEI skills, TEI does seem to be impacted, resulting in atypical profiles among autistic adults (Montgomery et al., 2008).

### Trait Emotional Intelligence

Past research suggests a substantial overlap between core autistic characteristics and TEI, such that aspects of socioemotional functioning that often differ for autistic adults (e.g., communication, flexibility, emotional reactivity, etc.) are accounted for by differences in TEI (Montgomery et al., 2008; Petrides et al., 2011). The current body of work in this area addresses differences in TEI between autistic and non-autistic people, as well as between non-autistic males and non-autistic females, but little consideration has been given to the overlap between these two foci to explore sex differences in TEI within the autistic population. The literature base suggests that autistic people differ from non-autistic people in TEI such that autistic people are lower in TEI than their non-autistic peers (Boily et al., 2017; Petrides et al., 2011). These observed differences in TEI, but not AEI, between autistic and non-autistic individuals are likely attributable to the “double empathy problem,” which posits that non-autistic people fundamentally misunderstand the experiences of autistic people (Jones et al., 2023; Milton, 2012). The double empathy problem reflects that there is indeed a disconnect between autistic and non-autistic people, but that this does not occur solely or even primarily because of deficits on the part of autistic people, but rather due to internalized ableism that prevents non-autistic people from considering and comprehending the emotional and other lived experiences of autistic people (Cheang et al., 2024). As a result of this disconnect, and owing to the fact that non-autistic people are the more socially privileged group, stigmatizing views of autistic people have been propagated over many decades, leading to internalized feelings of deficiency with regard to autistic people’s perceived emotional skills, including emotional intelligence (Niles & Harkins Monaco, 2019; Radulski, 2022).

Furthermore, while substantial research has considered sex differences in TEI in samples of non-autistic adults, the results are mixed as to whether males (Shahzad & Bagum, 2012) or females (Joseph & Newman, 2010) are higher in TEI. Recent research with non-autistic adults suggests that TEI does not significantly differ between males and females but is stable over time and significantly relates to satisfaction in social relationships, further reinforcing the need to understand this area in autistic adults (Parker et al., 2021). Although autism is associated with impaired TEI (Robinson & Petrides, 2020), few studies have examined sex differences in TEI in autism. One such study found higher TEI in non-autistic adults than autistic adults, and in males relative to females across both groups (Petrides et al., 2011). While these results are a promising foray into an exploration of sex differences in TEI among autistic adults, more research is needed in this area.

## Alexithymia

Similarly, research on alexithymia has historically focused on differences between autistic people and non-autistic adults or between non-autistic males and non-autistic females, but not the intersection of autism and sex. Alexithymia refers to challenges with emotional awareness, especially difficulties recognizing and expressing one’s own emotions (Lane et al., 1996). A recent development in the field suggests that the differences in emotion processing that have historically been considered an integral component of autism may actually be accounted for by co-occurring alexithymia instead (Kinnaird et al., 2019). Support for this new understanding comes from findings demonstrating that co-occurring alexithymia, not autism, accounts for autistic adults’ reduced emotion recognition of both faces and vocalizations (Cook et al., 2013; Reichebacher et al., 2012; Bird & Viding, 2014; Cuve et al., 2021). Work from Gerber and colleagues (2019) provides further evidence of the relationship between autism and alexithymia by demonstrating that autistic adults rate themselves higher in alexithymia symptoms than non-autistic adults, and that alexithymia is related to the quantity of social interactions reported. A recent meta-analysis corroborated Gerber and colleagues’ (2019) finding and reported that autistic people across various age groups scored significantly higher on measures of alexithymia than non-autistic people (Kinnaird et al., 2019). Kinnaird and colleagues (2019) further showed that the autistic group had a markedly higher prevalence of alexithymia compared to the non-autistic group (49.93% versus 4.89%, respectively). Furthermore, although absent of formal autism diagnosis, recent research, including meta-analytic work, demonstrated that autistic traits were associated with a higher degree of alexithymia, and that males were more likely than females to demonstrate more autistic traits, as well as more alexithymia (Mendia et al., 2024; Zhao et al., 2024). In non-autistic samples, results are mixed with regard to sex differences. While several studies indicate that males have higher rates of alexithymia (i.e., more impairment) than females (Garaigordobil, 2013; Bagby et al., 1988; Lane et al., 1998), others do not reflect any significant sex differences (Pasini et al., 1992). Despite extensive work comparing alexithymia in autistic and non-autistic people, and comparing non-autistic males and females, possible sex differences between autistic people are presently unclear as no analyses have explicitly considered this intersection.

### Empathy

The study of empathy in autism is long-standing, and the field’s understanding is constantly evolving. The role of both cognitive empathy (i.e., the ability to understand another’s emotional experience; Davis, 1983), and affective empathy (i.e., one’s own emotional responses to the emotional experience of another person; Tone & Tully, 2014) have been examined in autistic populations. Furthermore, differences in these areas have consistently been viewed as inextricably bound to the socioemotional differences that are a core feature of autism (Mul et al., 2018) as well as interpersonal relationships, which are often unique in autistic people (Bos & Stokes, 2019).

Empathy has also been researched extensively in non-autistic samples, including explorations of sex differences. Research in this area reflects an advantage for females over males across both cognitive and affective empathy, although there is some evidence that these differences depend on the type of measurement (Baron-Cohen et al., 2003; Eisenberg & Lennon, 1983). Specifically, while self-report data tends to favor females over males, recent research using more objective, biological methods suggest that males and females do not significantly differ in their empathetic responses (Pang et al., 2023). Despite some discrepant results across report styles, theories nonetheless abound as to why such striking differences exist. Some prominent theories posit that biological factors such as prenatal hormones promote more empathy in females (Auyeung et al., 2009). One notable theory that came forth from the biological perspective on sex differences in empathy is the extreme male brain (EMB; Baron-Cohen, 2002) theory that dichotomizes empathy (E) and systemizing (S; Baron-Cohen & Wheelwright, 2004). According to EMB theory, while empathizing allows for the prediction of others’ behavior based on caring about how they feel, systemizing predicts behaviors via analysis of the overarching “rules” governing a system, and the construction of novel systems (Baron-Cohen et al., 2003; Baron-Cohen & Wheelwright, 2004). Research in this area indicates that males tend to be more systemizing while females tend to be more empathetic (Baron-Cohen, 2002).

Likewise, the extant research suggests significant differences in empathy between autistic and non-autistic people such that autistic people tend to be lower in cognitive empathy, but perhaps not affective empathy, than their non-autistic peers across the lifespan (Auyeung et al., 2009; Baron-Cohen & Wheelwright, 2004; Bos & Stokes, 2019). In addition to prolific work comparing empathy in non-autistic males and females as well as in autistic and non-autistic people, some work has specifically focused on the intersection of autism and sex with regard to empathy. Research considering sex differences in empathy across the lifespan found that autistic females were higher in empathy than autistic males (Schneider et al., 2013; Song et al., 2019; de Giambattista et al., 2021). Work from Baron-Cohen and colleagues (2014) further contextualizes these findings and suggests that while autistic adult females are higher in empathy than autistic adult males, these differences are less extreme than those observed between non-autistic adult females and males. Despite some increases in research exploring sex differences in empathy within the autistic population, more research is nonetheless needed to further explore this complex phenomenon. Although a few studies have been conducted regarding sex differences in empathy within the autistic community, this limited body of work needs both replication and corroboration in order to match the level of work being done in this area with non-autistic people. Furthermore, whereas past research has examined these constructs among either autistic or non-autistic adults in distinct samples, a strength of this current research is its exploration of sex differences across both autistic and non-autistic adults in the same sample.

There is currently a dearth of research about sex differences within the autistic population regarding TEI, alexithymia, and empathy. This analysis therefore seeks to address the resultant gap in the literature by offering much-needed insight into these interrelated factors and their impact on emotional processing and social communication in autism. Moreover, since autism research tends to focus primarily on the experiences of autistic children and adolescents and typically prioritizes males over females, this analysis could increase awareness about the experiences of these historically understudied groups (Howlin et al., 2015; Mandy & Lai, 2017).

### Current Study

The data utilized in this analysis was originally collected as part of a larger, brief intervention study looking at the effects of active emotion recognition on social cognition in autistic and non-autistic adults. This analysis seeks to address the current gap in the literature regarding sex differences in TEI, alexithymia, and empathy among autistic and non-autistic people. While some previous work has examined sex differences in these areas among non-autistic people, there is a paucity of research examining sex differences in autistic people, especially with regard to alexithymia. Since TEI, alexithymia, and empathy are all related to the communication differences that are considered a hallmark of autism, an examination of sex differences in these areas could add valuable information to the field’s ongoing exploration of autism’s heterogeneity. In addition, these findings may provide insight into which people in the autistic community might benefit most from interventions intended to provide support in these areas and aid in the development of relevant treatment targets. There is presently a dearth of research regarding autistic adults, and particularly about autistic adult women, so the current research will substantiate a burgeoning body of work in this historically understudied area. Additionally, this research takes the unique approach of examining sex differences across both autistic and non-autistic adults in the same sample, rather than utilizing a solely autistic or non-autistic group as has been the precedent in this literature to date.

## Hypotheses

Based on previous research about sex differences in TEI, alexithymia, and empathy in non-autistic samples, as well as work comparing autistic and non-autistic adults in these areas, it was hypothesized that more autistic characteristics would be associated with less TEI, more alexithymia, and less empathy. It was further hypothesized that these relationships would be more prominent amongst males than females. In sum, we hypothesize that:

1. Autistic characteristics will be significantly related to various constructs.
  - Autistic characteristics will be negatively correlated with TEI.
  - Autistic characteristics will be positively correlated with alexithymia.
  - Autistic characteristics will be negatively correlated with empathy.
2. When considered concurrently, each of these constructs will emerge as significant predictors of autistic characteristics, and TEI will account for the most unique variance in the outcome variable.
3. Sex differences will be observed in the aforementioned domains such that the delineated relationships are more robust among males than females (i.e., males will display higher levels of alexithymia but lower levels of both TEI and empathy).

## Methods

### Participants

The sample consisted of 110 autistic and non-autistic adults (52 females; 98.90% cisgender; *M*_age_ = 22.80, *SD*_*age*_*= 6.34;* 43.6% White; *M*_*IQ*_ =102.82, *SD*_*IQ*_ = 15.43; see Table 1) taken from a larger intervention study (see Table 1 for further demographic information). Of these 110 participants, 86 completed measures and provided data for the domains of interest. Data collection occurred from 2014 to 2019. Non-autistic adults were recruited via the psychology department at a public university in the Northeastern region of the United States. Autistic adults were recruited via the same university, as well as local providers, autism-related events, and word of mouth from community contacts. Unlike many other areas within autism research that tend to focus on younger groups, examination of these interrelated constructs in adult autistic samples might be especially pertinent as normative changes across development might otherwise obscure the relationships. Participants received either course credit or a $25 Amazon gift card for their participation. All participants were over the age of 18 and provided informed consent prior to beginning the study. All participants spoke fluent English.

**Table 1:** Participant demographic information

Autistic (*n* = 50)	
Characteristic	Number (Percentage) or Mean (SD)
Sex	
Male	34 (70.8%)
Female	14 (29.2%)
Race	
White	27 (73%)
Black/African American	4 (10.8%)
Asian/Asian American	1 (2.7%)
Hispanic/Latino	1 (2.7%)
Native American/American Indian/Alaskan Native	1 (2.7%)
Native Hawaiian/Pacific Islander	1 (2.7%)
Other	1 (2.7%)
Age	
Mean	25.51 (7.64)
Range	18.36–45
Autism Severity (ADOS-2)	
Mean	6.29 (2.76)
Range	1–10
Overall IQ (KBIT-2)	
Mean	104.49 (18.77)
Range	48–136
**Non-Autistic (** ***n*** ** = 54)**	
**Characteristic**	**Number (Percentage) or Mean (SD)**
Sex	
Male	15 (28.3%)
Female	38 (71.7%)
Race	
White	21 (39.6%)
Black/African American	4 (7.5%)
Asian/Asian American	17 (32.1%)
Hispanic/Latino	11 (20.8%)
Other	1 (1.9%)
Age	
Mean	21.16 (4.79)
Range	18.14–47.71
Overall IQ (KBIT-2)	
Mean	101.69 (12.79)
Range	79–128

*Note. N* = 104

ADOS-2, Autism Diagnostic Observation Schedule – Second Edition; KBIT-2; Kaufman Brief Intelligence Scale – Second Edition

### Sample Characterization

Group assignment was determined using the Autism-Spectrum Quotient (AQ; Baron-Cohen et al., 2001) and the Autism Diagnostic Observation Schedule – Second Edition (ADOS-2; Lord et al., 2012). All participants completed the AQ, and those who met criteria for autism based on their AQ score completed an ADOS-2 Module 4, which is appropriate for adults over the age of 16 with fluent speech abilities. All ADOS-2 examiners were certified research-reliable in administration and scoring of the assessment prior to administration. Based on this method, 34 males were classified as autistic, as were 14 females, while 15 males and 38 females were classified as non-autistic.

### Measures

#### Kaufman Brief Intelligence Test – Second Edition (KBIT-2)

The KBIT-2 is a brief measure of intelligence (Kaufman & Kaufman, 2004). It is intended for use with individuals aged 4–90 and can be administered by non-psychologists. Standardized scores are calculated using age-specific norms. The IQ composite is composed of a verbal and nonverbal component. Graduate students and undergraduate research assistants were thoroughly trained prior to administering the KBIT-2 and received periodic performance evaluations to ensure that all assessment procedures were followed properly. All participants completed the KBIT-2.

### The Social Responsiveness Scale – Second Edition (SRS-2)

The SRS-2 is a 65-item questionnaire measuring autistic characteristics (Constantino & Gruber, 2012). This study specifically utilized the self-report version of the SRS-2, which is intended for use with individuals 19 years and older. Each item is rated on a 4-point Likert scale ranging from “Not True” to “Almost Always True. Scores can also be determined for each of the SRS-2’s five subscales: Social Awareness, Social Cognition, Social Communication, Social Motivation, and Restricted Interests and Repetitive Behavior. Each of the five subscales, as well as the overall total score, produces a standardized T-score, such that higher scores suggest greater impairment in the associated area. A standardized score of 60 or more on the SRS-2 is typically indicative of autism, with higher scores indicating more autistic characteristics. However, rather than consider autism as a binary variable (autistic vs. non-autistic), it will instead be conceptualized as a continuous variable (fewer autistic characteristics to more autistic characteristics).

### The Toronto Alexithymia Scale (TAS-20)

The TAS-20 is a self-report measure of alexithymia symptoms (Bagby et al., 1994). The TAS-20 consists of 20 items such as, “I have feelings that I can’t quite identify.” Each item is rated on a five-point scale ranging from “Strongly Disagree” to “Strongly Agree.” A raw total is calculated by summing the total ratings across items. Higher total raw scores suggest more alexithymia, and therefore larger differences in emotional awareness. In addition to an overall score, scores can also be calculated for each of three subscales: difficulty describing feelings, difficulty identifying feelings, and externally-oriented thinking. The TAS-20 has historically been used to gauge autistic adults’ alexithymia (Cook et al., 2013; Shah et al., 2016), and the measure has demonstrated superior test-retest reliability (*r* = .92) and convergent validity (*r* = .77) in that population compared to other measures of alexithymia (Berthoz & Hill, 2005). Furthermore, this measure demonstrated satisfactory internal consistency (Cronbach’s *α* = 0.87) in the current sample.

### The Basic Empathy Scale in Adults (BESA)

The BESA is a self-report measure of cognitive and affective empathy (Jolliffe & Farrington, 2006). Empathy is defined as “the understanding and sharing in another’s emotional state or context” (Cohen & Strayer, 1996). The BESA is comprised of 20 items such as, “I get caught up in other people’s feelings easily.” Each item is rated on a five-point Likert scale ranging from “Strongly Disagree” to “Strongly Agree.” In addition to the total score, scores on cognitive and affective empathy scales can be calculated by dividing the total score by the number of responded items included in each scale. The BESA has demonstrated good construct validity (*r* > .43) and internal consistency (Cronbach’s *α* = 0.81) and is considered a psychometrically sound measure of empathy (Jolliffe & Farrington, 2006; McLaren et al., 2019; Cabedo-Peris et al., 2021). Furthermore, this measure demonstrated satisfactory internal consistency (Cronbach’s *α* = 0.86) in the current sample.

### Schutte Emotional Intelligence Scale (SEIS)

The SEIS is a self-report measure of emotional intelligence, specifically trait emotional intelligence (Schutte et al., 1998). The measure is comprised of 33 items, such as “I can tell how people are feeling by listening to the tone of their voice.” Each item is rated on a five-point Likert scale from “Strongly Disagree” to “Strongly Agree.” A total score is derived by summing the item responses such that higher scores indicate more emotional intelligence. Notably though, since TEI “deficits” in autism may in fact be attributable to the double empathy problem, lower scores on the SEIS should be conceptualized as lower *perceived* emotional intelligence, and vice versa (Cheang et al., 2024; Jones et al., 2023; Milton, 2012). The SEIS also has three subscales in addition to the overall score: appraisal and expression of emotion, regulation of emotion, and utilization of emotion. The SEIS assesses perception, understanding, expression, regulating, and harnessing of emotion in the self and in others. This measure has demonstrated sound psychometric properties, including test-retest reliability (*r* = .78), internal consistency (Cronbach’s *α* > 0.70), and construct validity (*r* > .41; Jonker & Vosloo, 2008). Furthermore, this measure demonstrated satisfactory internal consistency (Cronbach’s *α* = 0.93) in the current sample.

### Data Analytic Plan

All analyses were conducted using SPSS software (Version 27; IBM Corp., 2020). Level of autistic characteristics (SRS-2) was the outcome variable of interest, while TEI (SEIS), alexithymia (TAS-20), and empathy (BESA) were the predictors. Furthermore, biological sex was utilized as a potential moderator of any significant relationships between the predictor variable and outcome variables. Notably, rather than considering autism as a binary variable (i.e., autistic or non-autistic), autistic characteristics were instead conceptualized as a continuous variable to allow for more nuanced analyses and interpretations aimed to help capture the heterogeneity of autism.

Prior to analyses to fulfill the study aims, a series of preliminary analyses were conducted. Analyses were conducted to assess whether relevant statistical assumptions (i.e., independence, normality, homoscedasticity, and absence of multicollinearity) were met. Independence of observations was assumed based on data collection procedures and was further assessed using the Durbin-Watson statistic, with values between 1.5 and 2.5 deemed acceptable. The assumption of normality was assessed visually via P-P plot. The assumption of homoscedasticity was assessed visually via a scatterplot of the residuals. Multicollinearity was assessed via examination of the variance inflation factor (VIF) value and the tolerance value associated with each variable in the analysis. The data was also assessed to determine whether multivariate and univariate outliers were present. Descriptive statistics for all variables in the analysis were computed.

To test Hypotheses 1a-1c, that autistic characteristics are positively correlated with alexithymia, but negatively correlated with TEI and empathy, bivariate relationships between all variables were assessed using Pearson’s correlations.

To test Hypothesis 2, a multiple linear regression was conducted to predict autistic characteristics simultaneously from the constructs of interest. As such, the model included participants’ total scores for the SEIS, TAS-20, and BESA as predictors of SRS-2 scores.

To test Hypothesis 3, moderation analyses were conducted to explore the role of biological sex in these relationships, to examine whether the relationships observed across these domains will be more robust among males than females. Moderation analyses were conducted via PROCESS plug-in in SPSS.

Based on the pattern of correlations reflected at the scale level, subsequent exploratory analyses examined differences at the subscale level of the SRS-2. These exploratory analyses probed for specific relationships between the subscales of the outcome variable (autistic characteristics) and the predictor variables (TEI, alexithymia, and empathy). To accomplish this goal, a multiple regression was repeated for each SRS-2 subscale, with SEIS, TAS-20, and BESA as predictors.

## Results

Having established that all assumptions (independence, normality, homoscedasticity, multicollinearity, absence of outliers) were met, Pearson product-moment correlations revealed large and significant relationships between level of autistic characteristics and TEI (*r* = -.81; *p* < .001), alexithymia (*r* = .71; *p* < .001), and empathy (*r* = -.52; *p* < .001), respectively (see Table 2). Due to the scoring of the SEIS, TAS-20, and BESA, this means that more autistic characteristics are associated with lower TEI, higher alexithymia, and lower empathy - indicating more challenges across these areas. Independent samples t-tests were utilized to explore overall sex differences in the areas of interest. Results utilizing the entire sample (*N* = 102) indicate that, compared to males (*M* = 116.74, *SD* = 24.54), females (*M* = 127.64, *SD* = 14.41) experience significantly higher TEI, *t*(83) = 2.55, *p* = .013. Similarly, compared to males (*M* = 70.05, *SD* = 11.93), females (*M* = 76.53, *SD* = 9.03) experience significantly higher empathy, *t*(83) = 2.85, *p* = .006. However, no significant sex differences emerged with regard to alexithymia, *t*(83) = -1.44, *p* = .15.

**Table 2:** Bivariate correlations between variables of interest

Measure	1	2	3	4
1. SRS-2 Total Score	1	− 0.81***	0.71***	− 0.52***
2. SEIS Total Score	–	1	− 0.72***	0.65***
3. TAS-20 Total Score	–	–	1	− 0.58***
4. BESA Total Score	–	–	–	1

*Note* BESA, Basic Empathy Scale in Adults; SEIS, Schutte Emotional Intelligence Scale; SRS-2, Social Responsiveness Scale, Second Edition; TAS-20, Toronto Alexithymia Scale

***Correlation is significant at the 0.001 level (2-tailed)

Additionally, two-way ANOVAs were conducted to examine sex differences in the areas of interest between autistic and non-autistic participants. We first evaluated the effects of autism and sex on TEI. The results indicated a significant main effect for autism, *F*(1, 81) = 23.23, *p* < .001, partial η2 = 0.22, such that autistic participants demonstrated lower TEI than non-autistic participants. However, no significant main effect emerged for sex, *F*(1, 81) = 0.56, *p* = .46, partial η2 = 0.007; and there was no significant interaction between autism and sex, *F*(1, 81) = 1.05, *p* = .31, partial η2 = 0.013. Next, we evaluated the effects of autism and sex on empathy. The results indicated a significant main effect for autism, *F*(1, 81) = 9.63, *p* = .003, partial η2 = 0.106, such that autistic participants demonstrated lower empathy than non-autistic participants. However, no significant main effect emerged for sex, *F*(1, 81) = 1.65, *p* = .20, partial η2 = 0.02; and no significant interaction was found between autism and sex, *F*(1, 81) = 0.04, *p* = .84, partial η2 = 0.001. Finally, we evaluated the effects of autism and sex on alexithymia. The results indicated a significant main effect for autism, *F*(1, 81) = 18.60, *p* < .001, partial η2 = 0.94, such that autistic participants demonstrated higher alexithymia than non-autistic participants. However, no significant main effect emerged for sex, *F*(1, 81) = 0.03, *p* = .87, partial η2 < 0.001; and no significant interaction was found between autism and sex, *F*(1, 81) = 1.14, *p* = .29, partial η2 = 0.014.

The overall model for multiple regression, which included TEI, alexithymia, and empathy, was statistically significant (*p* < .001), accounting for 69.2% of the variance in total SRS-2 score. Total SEIS score was a significant predictor of total SRS-2 score (*B*=-0.38, *p* < .001; see Table 3), suggesting that more autistic characteristics are predicted by lower SEIS total score, and therefore lower levels of TEI. Total TAS-20 score also emerged as a significant predictor of total SRS-2 score in this model (*B* = 0.26, *p* = .003), suggesting that more autistic characteristics are associated with higher TAS-20 scores, and therefore higher levels of alexithymia. However, total BESA score was not a significant predictor of SRS-2 score in this model (*B* = 0.08, *p* = .40). In sum, only SEIS total score and TAS-20 total score emerged as significant predictors.

**Table 3:** Results of multiple regression model predicting total SRS-2 score (*N* = 86)

Predictor		B	SE
Constant		87.54***	10.68
SEIS Total Score		− 0.38***	0.06
TAS-20 Total Score		0.26**	0.09
BESA Total Score		0.08	0.09

*Note*. BESA, Basic Empathy Scale in Adults; SEIS, Schutte Emotional Intelligence Scale; SRS-2, Social Responsiveness Scale, Second Edition; TAS-20, Toronto Alexithymia Scale

** Correlation is significant at the 0.01 level (2-tailed)

*** Correlation is significant at the 0.001 level (2-tailed)

**Fig. 1:**
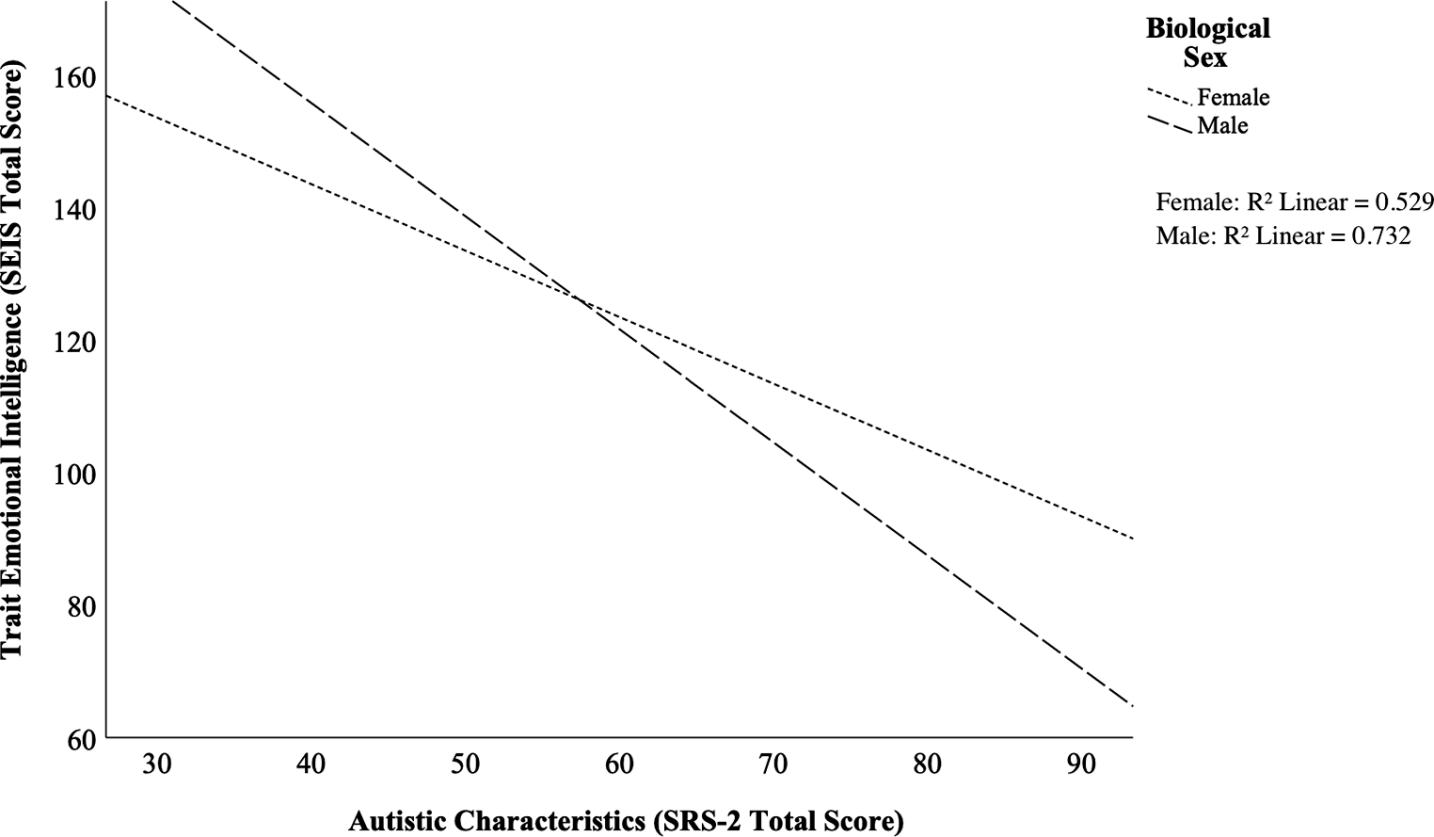
Relationship Between SRS-2 and SEIS Moderated by Sex. Differences were seen between males and females in the relationship between trait emotional intelligence and autistic traits. Specifically, while a negative relationship emerged between trait emotional intelligence and autistic traits, this relationship was particularly prominent amongst males. *Note.* SEIS, Schutte Emotional Intelligence Scale; SRS-2, Social Responsiveness Scale, Second Edition. ***. Correlation is significant at the 0.001 level (2-tailed)

Sex moderated the relationship between autistic characteristics and TEI (*B*=-0.70, *p* = .002). These results indicate that higher levels of autistic characteristics were associated with lower TEI more so in males than in females (see Fig. 1). Similar moderation analyses were conducted to explore the role of sex on the relationship between autistic characteristics and alexithymia (*p* = .26), and between autistic characteristics and empathy (*p* = .022), but the results were non-significant (*B* = 0.20, *p* = .26).

## Discussion

Consistent with prior research, sex differences emerged with regard to TEI and empathy in the entire sample of autistic and non-autistic adults. Additionally, our preliminary analyses reflect that autistic adults experience lower levels of TEI and empathy, as well as higher levels of alexithymia, than their non-autistic peers. In tandem, these findings indicate nuanced relationships once autism and sex are considered concurrently in these relationships. However, the current study was the first study in adults that explored the individual and concurrent relationships between autistic characteristics and TEI, alexithymia, and empathy, respectively, rather than consider autism as a binary construct. Additionally, these analyses sought to explore what role sex might play in these relationships. Our results indicate that autistic characteristics were associated with higher rates of alexithymia, lower TEI, and lower empathy. Furthermore, we found that more autistic characteristics were predicted by lower self-reported TEI, as well as greater alexithymia. However, empathy did not significantly predict autistic traits in this adult sample when controlling for TEI and alexithymia. Furthermore, additional analyses examining the role of sex on these relationships revealed that the relationship between autistic traits and TEI was more pronounced amongst males than females, indicating that males with more autistic characteristics may be especially at risk for discounting their TEI abilities. Alternatively, sex did not appear to substantially moderate the relationships between autistic traits, alexithymia, and empathy, respectively.

Overall, consistent with our hypotheses, findings suggest that adults with more self-reported autistic characteristics have more difficulty interacting with emotion-laden information, recognizing and interpreting their own emotional states, and understanding and responding to others’ emotional states. These findings align with prior research in these areas positing that lower TEI, heightened alexithymia, and lower empathy are more common in autistic people and are associated with the differences in social communication that are considered a hallmark of autism (Boily et al., 2017; Gerber et al., 2019; Petrides et al., 2011; Kinnaird et al., 2019; Quinde-Zlibut et al., 2021). Whereas these constructs have been considered across the lifespan, including adulthood, most of the work to this point has considered autism as a binary, largely based on clinical diagnosis. Rather than considering autistic characteristics as a binary variable, it was instead conceptualized as a continuous variable to allow for more nuanced analyses and interpretations. The current study’s choice to consider autism as a continuum and to therefore explore differences across these domains based on level of autistic characteristics is unique. Results from this analysis indicate that adults with more autistic characteristics, regardless of diagnostic status, demonstrate differences in TEI, alexithymia, and empathy. As our collective understanding of autism grows, nuanced approaches that consider number of level of characteristics associated with autism may provide valuable insights. Furthermore, given the trend for some adults, especially females or those from ethnoracial minority groups, to self-diagnose autism due to systematic barriers around formal diagnosis, taking this approach may incorporate the experiences of autistic people who would otherwise be assumed non-autistic in research (Lewis, 2016; Sarrett, 2016; Zener, 2019). Among this sample of autistic and non-autistic adults, it appears that these relationships are robust, thus adding to the literature base regarding these constructs in autism.

Prior literature has not examined these three constructs concurrently, and the current findings suggest that TEI, alexithymia, and empathy may play differential roles in predicting autistic characteristics, warranting further exploration. When considering TEI, alexithymia, and empathy concurrently, TEI and alexithymia uniquely predict autism characteristics above and beyond other variables of interest. Specifically, empathy was not a significant predictor of autistic characteristics when accounting for TEI and alexithymia. This finding may be especially pertinent in light of the decades of research suggesting that autistic people lack empathy (Sigman et al., 1992; Charman et al., 1997). This assertion about autistic people’s capacity for empathy has had deleterious effects and could increase stigma toward this community. The current analysis may offer additional context to the evolving understanding of empathy and autism by suggesting that TEI and alexithymia could account for the differences in empathy historically observed in this population. As such, future research should delve deeper into these constructs to determine whether measures that seemingly reflect deficits in empathy among autistic people could actually be measuring related constructs such as TEI and alexithymia. Consequently, such work could help to undo stigmatizing societal beliefs related to lack of empathy in autism and potentially promote broader acceptance of autism.

In addition to these initial relationships, of particular relevance to this analysis was the role that sex plays in these relationships, as sex differences across these domains has thus far been underexplored in autism despite extant knowledge that the autistic phenotype differs between males and females (Ferri et al., 2018; Werling & Geschwind, 2013). Only the relationship between autistic characteristics and TEI was moderated by sex, and this relationship was in the hypothesized direction that males with more pronounced autistic characteristics have lower TEI than males with less autistic characteristics or females with more autistic characteristics. Although the relationships are complex, this finding aligns most with previous work conducted with non-autistic adults which suggested that females have higher TEI than males (Johnson & Newman, 2010). Given that TEI reflects self-perceptions of emotional abilities, the observed differences may be indicative of socialization differences between males and females in neurotypical populations (Sanchez-Nunez et al., 2008). Ergo, since females may be more likely than males to be positively reinforced for engaging with and utilizing emotional information, they may also rate themselves more highly in their ability to do so. It appears that this sex difference persists in autistic populations despite overall reduced TEI among autistic adults. Autistic females seemingly receive conflicting socialization around emotional intelligence such that they are expected to have greater capacity in this area due to their sex while simultaneously being expected to have some level of impairment due to their autism (Chaidi & Drigas, 2020; Garaigordobil, 2020; Sanchez-Nunez et al., 2008). Based on the pattern of results reported herein, it seems that social learning and reinforcement around autism may be more salient or influential than social learning and reinforcement around sex such that autistic females rate their TEI abilities higher than autistic males but lower than non-autistic males.

On the other hand, the relationships between level of autistic characteristics and alexithymia and between autistic characteristics and empathy were not significantly moderated by sex, indicating that males and females may experience similar trends in these areas. Given the present disunity related to sex differences in alexithymia among adults, it is not wholly unsurprising that no significant sex differences emerged in the current study. This finding could indicate that meaningful sex differences may not exist among autistic and non-autistic adults in this area, but additional work is needed to approach this conclusion more conclusively. Alexithymia is an area of growing interest, particularly in autism research, and increased knowledge may elucidate the role of sex in the future. On the other hand, the lack of sex differences related to empathy is somewhat surprising given past findings that autistic females are higher in empathy than autistic males. However, there is very limited research addressing the intersection of sex and autism related to empathy, so more work is needed to further clarify this relationship. It is possible that the lack of sex differences observed related to empathy in the current study is related to the conceptualization of autism as a continuum rather than a binary, since prior studies have considered sex differences in empathy based on autism diagnosis, which could indicate that this interaction only exists within the binary of autism diagnostic status rather than in the continuum of autism more broadly. Furthermore, the extant literature regarding both alexithymia and empathy reflects inconsistent findings related to sex differences, so it is possible that heretofore unconsidered or latent factors other than sex (e.g., gender, culture) underlie differences in these domains.

In sum, the results from the current study indicate that that adults with higher levels of self-reported autistic traits may experience more difficulties in successfully integrating and utilizing affective information, characterizing their own emotional states, and responding to the emotional needs of others. When considering TEI, alexithymia, and empathy concurrently, TEI and alexithymia predict autism characteristics above and beyond other factors, making them key areas for further exploration. Moreover, it appears that sex plays a role in the relationship between autistic characteristics and TEI such that differences are especially prominent for males. As such, males with more self-reported autistic characteristics may experience more challenges related to proper use of emotional information (e.g., inappropriate use of humor, apathetic response to challenging or upsetting circumstances) than their non-autistic peers or than autistic females. Therefore, targeted supports to build TEI skills are likely to benefit all people with high levels of autistic characteristics, but such supports may be especially crucial for males.

### Clinical Implications

In light of the established relationships between these domains and differences in social communication, TEI, alexithymia, and empathy could each be considered key treatment targets for autistic adults. Intervening to promote growth in these underlying areas could facilitate changes in social communication, thereby assisting autistic adults in establishing social relationships and building supportive communities for themselves. This may be especially important since loneliness or the absence of such supportive communities has been linked to poorer quality of life in this population (Mason et al., 2018; Lin & Huang, 2019). As such, targeted treatments to diminish alexithymia while honing TEI and empathy could prove beneficial for adults with more autism characteristics, regardless of whether they have a formal diagnosis. While some progress has been made toward developing evidence-based supports for autistic children and adolescents in these areas through in vivo practice during targeted games and Theory of Mind training, more work is needed to adapt these supports for autistic adults (Hassan et al., 2021; Holopainen et al., 2019).

Clinicians and other professionals who diagnose autism or work with autistic adults should note that while autistic females may report more positively on their TEI abilities and may indeed present as more competent in this area, they nonetheless need support in this domain. Professionals working with this population may need to refine their understanding of the autistic phenotype since females may mask their autistic characteristics and therefore by misdiagnosed and lack access to services as a result. Furthermore, tailored interventions for autistic adults, particularly males, related to TEI could facilitate positive changes in their social connectedness and overall quality of life by extension. More research is needed to further explore sex differences in various domains related to autism in order to facilitate the field’s understanding of different phenotypes with the ultimate goal of limiting misdiagnoses and missed diagnoses based on presentations that do not fit the stereotypical autistic presentation (e.g., a female with some ability to engage socially, utilize and respond appropriately to emotional information, and express concern for others can still be autistic but might be missed). Furthermore, greater nuance is needed in assessment tools and interpretive skills in order to account for these phenotypic sex differences in autism (Begeer et al., 2013; Giarelli et al., 2010 Kalb et al., 2022; Rynkiewicz et al., 2016). Additionally, these analyses provide some insight into support needs for clients more broadly, as results demonstrate that males in the whole sample displayed lower TEI and empathy than their female peers. In light of these findings, mental health providers working with a variety of presenting problems should be aware that males may benefit from additional skills and supports in these areas.

### Limitations and Future Directions

Despite the promising results reported herein, there were nonetheless a few notable limitations that warrant consideration. Firstly, increasing the sample size would improve the power and might allow for further exploration of these domains individually and concurrently. Furthermore, participants in the autism group were predominantly male, while participants in the non-autism group were predominantly female, meaning that the groups were not balanced for autism by sex. This imbalance likely impacted the results reported herein, and future research should seek to recruit more autistic female participants to contribute to more robust understanding of sex differences in autism. Secondly, this study’s participants were predominantly cisgender, and no participants identified as transgender despite the fact that autistic people are significantly more likely than their non-autistic peers to identify as transgender or otherwise gender diverse (Thrower et al., 2020). This facet of participant composition is especially important to note given that TEI and empathy are both related to gender norms and socialization. Thirdly, only two adults with an IQ below 70 were included in this sample, which limits the generalizability of findings to adults with intellectual disability. Autism is associated with a wide range of cognitive functioning, so it is possible that the relationships discussed herein might look quite different in autistic adults with lower cognitive functioning. Finally, the use of self-report measures may have affected the results if participants attempted to answer in socially desirable ways or if they had poor insight into their own abilities in these areas.

Future research in this area should therefore attempt to correct these identified limitations. Specifically, future researchers should consider collecting data from multiple informants, especially since self-report measures may not be the most reliable data source. Expanding the informants in subsequent work could provide valuable information about how autistic people perceive their abilities in these areas compared to how others perceive their abilities. Future researchers might also consider the role of gender on TEI, alexithymia, and empathy rather than sex. In the present student, participants’ gender identity overwhelmingly aligned with their sex assigned at birth, but interested parties might effortfully recruit more gender-diverse samples and consider whether gender identity moderates these relationships. Since both TEI and alexithymia remained significant predictors of SRS-2 score when controlling for one another, the interplay between these domains is ripe for further consideration in autistic people.

Aside from these limitations, this study has practical significance in identifying which interventions may most benefit autistic adults. Based on these findings, it seems that developing interventions that target skills related to TEI and alexithymia might prove quite beneficial to autistic adults. Such interventions could improve recognition of their own emotions and functioning in the social world. Furthermore, the exploration of sex differences adds valuable information to this area by suggesting that additional TEI support should be allocated to autistic males. Overall, these findings contribute to a growing body of work regarding adult outcomes and support the provision of lifespan support to autistic people.

## Footnotes

1 The language utilized herein is congruent with suggestions from Bottema-Beutel and colleagues (2021) in the hopes of limiting stigmatization. These suggestions include, but are not limited to, the use of identity-first rather than person-first language, non-medicalized language (e.g., minimizing pathologizing language such as “disorder”), and references to “non-autistic” people rather than “typically-developing” or “neurotypical” people. However, when referencing the specific DSM-5 disorder rather than the person, Autism Spectrum Disorder (ASD) will be used.
