# Correction to: The Moderating Effect of Sex on Autistic Trait Emotional Intelligence, Alexithymia, and Empathy

**DOI:** 10.1007/s10803-024-06619-5

**Published:** 2024-10-24

**Authors:** Mary Isaac Cargill, Matthew D. Lerner, Erin Kang

**Affiliations:** 1 Psychology Department, Montclair State University, 1 Normal Ave, Montclair, NJ 07043 USA; 2 AJ Drexel Autism Institute, Drexel University, 3020 Market Street Suite #560, Philadelphia, PA 19104 USA

**Correction to: Journal of Autism and Developmental Disorders (2024)**

10.1007/s10803-024-06540-x

In this article in the reference “Mendia, J., Zumeta, L. N., Cusi, O., Pascual, A., Alonso-Arbiol, I., Díaz, V., & Páez, D. (2024). Gender differences in Alexithymia: Insights from an Updated Meta-Analysis. Personality and Individual Differences, 227, 112710. 10.1016/j.paid.2024.112710 the DOI missed and incorrectly appeared as “Mendia, J., Zumeta, L. N., Cusi, O., Pascual, A., Alonso-Arbiol, I., Díaz, V., & Páez, D. (2024). Gender differences in Alexithymia: Insights from an Updated Meta-Analysis. Personality and Individual Differences, 227, 112710.”

The original article has been corrected.

